# Practice variation across consent templates for biobank research. a survey of German biobanks

**DOI:** 10.3389/fgene.2013.00240

**Published:** 2013-11-14

**Authors:** Irene Hirschberg, Hannes Knüppel, Daniel Strech

**Affiliations:** Hannover Medical School, Institute for History, Ethics and Philosophy of Medicine / Centre for Ethics and Law in the Life SciencesHannover, Germany

**Keywords:** biobank, tissue bank, broad consent, informed consent, survey, thematic text analysis, research ethics

## Abstract

**Introduction: **Informed, voluntary, and valid consent from biomaterial donors is a precondition for biobank research. Valid consent protects donors’ rights and helps maintain public trust in biobank research. Harmonization of consent procedures in biobank research is needed, because of the widely shared vision on national and international networking of biobanks including data and sample sharing. So far, no study has assessed and compared the content of current consent forms especially for biobank research. The objective of this study was to perform a content analysis of consent forms in German biobanks.

**Methods: **Based on ten guidelines for biomedical research, we developed an assessment matrix with 41 content issues that are potentially relevant for consent forms in biobank research. This assessment matrix was applied in a thematic text analysis to 30 consent documents of German biobanks identified via the German Biobank Registry in July 2012.

**Results: **Coverage of the 41 items in the assessed consent forms varied widely. For example, the items “Right to withdraw consent (without disadvantage),” “Policy for genetic information/consent to genetic analyzes” and “International cooperation/transborder use” were addressed in 97, 40, and 23% of all 30 consent forms respectively. The number of items covered by a single consent form ranged from 9 to 36 (22–88% out of 41 items).

**Discussion: **Our findings serve as a starting point to reflect upon the spectrum of consent issues that must be addressed in biobank research. The findings show that the majority of consent forms for German biobanks, if not all, should be improved and harmonized to better support an informed and balanced choice of potential donors and to facilitate networking of biobanks. Best practice models for consent forms in biobank research should be developed and biobank operators need to be more aware of relevant consent issues.

## INTRODUCTION

Biobanks are collections of human biological samples and related health/personal information. A high quality biobank that organizes samples for use by biomedical researchers is seen as an important resource for health research, including basic research, questions in personalized or stratified medicine (genetic and other biomarkers), and research in widespread diseases (Asslaber and Zatloukal, 2007; Zika et al., 2010).

The development of large-scale population-based as well as disease-specific biobanks also implies several new ethical, legal and social challenges. These comprise, e.g., issues around the role of ethics committees, data protection, dealing with incidental findings, public involvement measures, and particularly regarding the need for new or at least modified models of informed consent of the donors (Budimir et al., 2011; Gottweis and Kaye, 2012). As acquired for diagnostic and therapeutic procedures in clinical as well as in research settings, informed, voluntary, and valid consent is a precondition for the collection of samples and clinical data of donors in a biobank. It also tends to protect donors’ rights and autonomy and to maintain public trust in biobank research. Crucial points include the scope and content of consent forms, for example, how do consent forms address potential future research projects that might involve the donated biomaterials (broad/general vs. narrow/specific consent) (Cambon-Thomsen et al., 2007; Greely, 2007; Hansson, 2009; OECD, 2009; Budimir et al., 2011; Deutscher Ethikrat, 2011). Additionally, biobank research consent procedures and documentation involve different or additional aspects compared with clinical research, e.g., concerning data protection and data sharing with other researchers (Hoeyer et al., 2005; Beskow et al., 2010; McGuire and Beskow, 2010; Pawlikowski et al., 2011). Besides, the harmonization of consent templates, at least of the most important criteria, is essential for future cooperation and networking at the national and international level.

Though information and consent documents do certainly not substitute the discussion between clinician/researcher and patient/participant, they are an important component within the informed consent procedure and its documentation, as well as for legal aspects. Regarding quality and performance of consent forms in clinical or research contexts, several empirical studies demonstrated that consent forms are often not adequate in content, comprehensibility or practicability and do not meet participants’ needs. Improvements are necessary within consent procedure and documents to support an adequately well-balanced and evidence-based decision-making process by participants (Jefford and Moore, 2008; Padhy et al., 2011; Brehaut et al., 2012; Mandava et al., 2012).

Several sets of ethical guidance define the required criteria for consent in clinical research, e.g., (Council for International Organizations of Medical Sciences [CIOMS], 2002; World Medical Association [WMA], 2008). Some guidelines also explicitly mention required criteria for consent in biobank research, e.g., (OECD, 2009). Nevertheless, there is no specific guidance for biobank research and consent procedures that can be employed as a tool for the assessment of consent forms’ content in clinical or biobank research. Furthermore, we currently lack a broadly accepted “best practice” model for consent forms in biobank research (Gottweis and Kaye, 2012).

Also in Germany, the number and importance of biobank facilities and projects with similar requirements is increasing, e.g., in population-wide studies such as KoraGEN and PopGEN, and the National Biobank Initiative (“Nationale Biomaterialbanken Initiative”) funded by the German Federal Ministry of Education and Research (Bundesministerium für Bildung und Forschung, BMBF).

Existing studies that describe different biobanks and their governance strategies also indicate challenges in consent procedures (Hirtzlin et al., 2003; Zika et al., 2010; Gottweis and Kaye, 2012). Some research groups propose a unified consent model or possible content for a consent form in biobank research (Porteri and Borry, 2008; Salvaterra et al., 2008) or for whole genome sequencing studies in the clinical context (Ayuso et al., 2013). Besides, there are studies comparing participant information and consent forms in genetic research (Mascalzoni et al., 2010) or in clinical trials (Hüppe et al., 2013). But so far, no study has assessed and compared the content of consent forms for biobank research. The objective of this study was to perform a survey and content analysis of currently used consent forms in German biobanks.

## MATERIALS AND METHODS

To improve the comprehensiveness, reliability, validity, and objectivity of our assessment of biobank specific consent forms, we first systematically developed an assessment matrix of potentially relevant issues that might be addressed in consent documents for biobank research. We then applied this assessment matrix to a sample of consent forms from German biobanks.

### DEVELOPMENT OF THE ASSESSMENT MATRIX

To identify issues that might be addressed in consent documents for biobank research, we referred to frequently cited policies, guidelines, and regulations dealing with general recommendations for consent procedures in biomedical research (mainly clinical studies) and/or biobank research. More specifically, we used nine prominent guidelines for biomedicine (Council of Europe, 1997), biomedical research with humans in general (European Parliament and the Council of the European Union, 2001; Council for International Organizations of Medical Sciences [CIOMS], 2002; European Medicines Agency [EMEA], 2002; Council of Europe, 2005; World Medical Association [WMA], 2008; US Department of Health and Human Services, 2009), and biobank or biomaterial research in particular (Council of Europe, 2006; OECD, 2009). For the national (German) context, we referred to a “commented checklist” of issues in patient information and consent for clinical studies with an annex for biobank research (Harnischmacher et al., 2006). The set of selected guideline is a purposive sample. Most of the chosen guidelines are, for instance, indicated as important legal and ethical frameworks in other sources such as the Oxford Textbook of Clinical Research Ethics (Capron, 2008; Emanuel et al., 2008). We have discussed the selection of guidelines and the resulting construct validity of the matrix of consent issues internally and with experts from the fields of law and biobank research.

In all ten documents, one author (Irene Hirschberg) searched for text passages that explicitly or implicitly mentioned issues of potential relevance to the content of consent forms in biomedical research in general, and applicable to biobank research. Examples mentioned as necessary information for consent included “general procedures and safeguards used to protect privacy and confidentiality,” the “policy with respect to benefit sharing” and “information on the human biological material and data to be collected, their intended uses, storage, transfer and their disposal technique” (OECD, 2009). An example for a relevant but implicitly mentioned issue dealt with the removal of biological materials after death: “biological materials should not be removed from the body of a deceased person for research activities without appropriate consent or authorisation.” (Council of Europe, 2006) Other issues were mentioned only in relation to the research protocol, such as “incentives for subjects” (World Medical Association [WMA], 2008),” or came under the discussion of policy, but indicated as relevant to participants: “The HBGRD’s policy should also address the situation where participants become legally incapacitated or die. It is essential that the HBGRD provide information on their policy to the participant or the appropriate substitute decision-maker at the time of consenting.” (OECD, 2009) The results and any ambiguity were discussed with the other authors (Hannes Knüppel and Daniel Strech). We excluded generally important aspects of biomedical research that were mentioned in one or more guidelines but were not considered relevant to the content of consent forms in biobank research, e.g., “trial treatment and random assignment (European Medicines Agency [EMEA], 2002)” and “alternative procedures or courses of treatment” (US Department of Health and Human Services, 2009). We also excluded formal aspects such as “title of the document” or “date/signature” (Harnischmacher et al., 2006).

We compared the mentions of issues relevant to consent forms in biobank research in different guidelines, and developed categories for similar mentions of issues. During the development of these categories, we slightly adapted the items arising from the clinical study context to the biobank context. For instance, we revised the wording “duration of participation in trial/study” to “duration of participation or storage.” In some cases we performed a synthesis of issues, e.g., subsuming “money or material goods” (Council for International Organizations of Medical Sciences [CIOMS], 2002), “payment” and “expenses” (European Medicines Agency [EMEA], 2002), “additional costs” (US Department of Health and Human Services, 2009), “incentives” (World Medical Association [WMA], 2008), and “allowance” (Harnischmacher et al., 2006) under “Payment/allowance and additional costs.” We finally obtained 41 items grouped under four main headings (see Results and **Table 1**). Each of the 41 items derives from at least two guidance documents.

**Table 1:** Representation of 41 consent issues for biobank research in 30 German consent documents.

Consent issues for biobank research	Origin: mention in guidelines for the regulation of biomedical research (*n* = 10)		Application: mention in biobank-specific consent documents (*n* = 30)	
Assessment items	*n*	%	*n*	%
**(A) General information**
Research explanation and purpose	10	100	28	93
Future development and changes	5	50	9	30
Biobank design and structure	5	50	21	70
Funding and (conflict of) interests	6	60	6	20
Duration of participation or storage	7	70	15	50
Biomaterial: types and quantity of specimen	3	30	27	90
Data: type and quantity of data	3	30	19	63
Description of collection procedures and additional tests	8	80	26	87
Sample collection: further examination needed/follow up-points	2	20	23	77
Rights/ownership of samples and data and their transfer	2	20	17	57
Opinion or approval of ethical review board/committee	5	50	16	53
**(B) Conditions of participation**
Dimension of consent: scope, safeguards and conditions	4	40	15	50
Free and voluntary participation	10	100	24	80
Right to withdraw or alter consent (without disadvantage)	10	100	29	97
Withdrawal: modalities and consequences regarding biomaterial and data	5	50	24	80
Decision on participation/withdrawal without affecting medical care or relationship to physician	5	50	15	50
Compensation and insurance cover	7	70	5	17
Options (partial consent)	3	30	16	53
**(C) Consequences of participation**
Direct benefit for participant	10	100	15	50
Indirect benefit for subgroups or society	10	100	19	63
Risk	10	100	21	70
Payment/allowance or additional costs	5	50	10	33
Benefit-sharing	3	30	18	60
Feedback on findings or incidental findings	6	60	20	67
Publication of data only unlinked	2	20	17	57
Re-contacting of participant: purpose and conditions	4	40	18	60
Contact person/point	5	50	18	60
**(D) Dealing with data and biomaterial**
Confidentiality of records and data/extent and limits of confidentiality	9	90	24	80
Privacy rights and procedures/safeguards, data processing, and identifiability of data and samples	6	60	27	90
Use of health data and records and their purpose	3	30	20	67
Storage of data and biomaterial	3	30	20	67
Policy for genetic information/consent to genetic analyses	2	20	12	40
Contact with or disclosure to participant’s physician	2	20	19	63
Policy on use/disclosure to third parties for non-research purpose	3	30	7	23
Sharing data and material with other researchers/policy and process	2	20	24	80
International cooperation/trans-border use	2	20	7	23
Commercialisation and collaboration with for-profit entities	5	50	17	57
Right of access to personal data	3	30	5	17
Disposal or destruction of data and material	3	30	21	70
Dealing with data and material after participants die or become incapacitated	2	20	0	0
Removal of material after death	2	20	1	3

### BIOBANK SURVEY AND ANALYSIS OF THE CONSENT DOCUMENTS

To get an overview of consent documents and templates currently used in biobank research in Germany, we considered all biobanks registered in the German Biobank Registry (“Deutsches Biobanken-Register,” ) in July 2012. We excluded six of the 108 registered biobanks because they were either doubly mentioned in the register or did not exist anymore (or their web site could not be accessed). Nine of the remaining 102 biobanks had consent forms publically available on their websites. We asked the heads of the other 93 biobanks via email to send us their consent documents. In this email we made clear that German biobanks will not be named individually in the dissemination of findings from our study. The response rate after repeated contact was 48% (44/90 biobanks, three mailing addresses were incorrect). Not all biobanks were willing to provide their consent document; some biobanks used a shared consent form, and two biobanks provided two documents for different purposes. At last, we were able to include 30 consent documents of 33 biobanks in our analysis. If the biobanks provided additional participant information and a consent form, we included both documents in the analysis.

The representation of all 41 items in each of the 30 consent documents was assessed according to standards in thematic text analysis (Dixon-Woods et al., 2005). All researchers were experienced in thematic text analysis and research ethics. The consent forms were read in full by two authors (Irene Hirschberg and Hannes Knüppel) independently to identify and extract text passages corresponding to each of the 41 consent issues for biobank research. For the purpose of this study the authors only rated whether the items were mentioned or not. For example, the authors did not evaluate in depth whether these items were sufficiently comprehensible. After the independent text extraction and rating the researchers compared their results. Discrepancies between the resulting spreadsheets were identified in 114 (9.3%) of all 1,230 ratings (41 ratings for each of the 30 consent forms). These discrepancies were discussed and resolved with the third author (Daniel Strech).

## RESULTS

### ASSESSMENT MATRIX

Our assessment matrix comprises 41 items in four categories: (A) “General information” covering, e.g., explanation of the type of research and its purpose, (B) “Conditions of participation” including background on voluntary participation, consent conditions and scope, (C) “Consequences of participation” comprising items on, e.g., risks and benefits, and (D) “Dealing with data and biomaterial” encompassing items on, e.g., data protection measures and cooperation with other parties. **Table 1** presents the 41 items of the assessment matrix for consent in biobank research, the number of guidance sets from which each item derived, and the extent to which the items were mentioned in the German biobank sample (see **Table 1**).

### GERMAN CONSENT DOCUMENTS FOR BIOBANK RESEARCH

Our sample comprised 30 consent documents from 33 German biobanks registered in the German Biobank Registry in July 2012. The sample includes different types of biobank with varying characteristics as type (population-wide or disease-specific biobank, or clinical study with sample collections), number of participants, organization and funding, and inclusion of healthy probands or patients (e.g., inclusion of all admitted patients). However, all registered biobanks are considered to perform biobank research. The German Biobank Registry is operated by the TMF (Technology, Methods, and Infrastructure for Networked Medical Research) and is funded by the German Federal Ministry of Education and Research.

Most of the biobanks had developed their own consent documents; one biobank just used a consent form for genetic diagnostic similar to a template of the German Society of Human Genetics. The consent documents (participant information and consent form) varied in several characteristics, e.g., in length of document (one to five pages), target group (patient, healthy participant, next kin, children, or parents), scope of consent, content, complexity and comprehensibility.

The coverage of the 41 items by the 30 consent documents was very variable. One item (“Right to withdraw or alter consent (without disadvantage)”) was addressed in 97%, another item (“Dealing with data and material after participants die or become incapacitated”) was not addressed by any consent form (see **Table 1**). Though for the interpretation of the results, it has to be considered that a few items – as “Removal of samples after death” – are not applicable to all biobanks. Nevertheless, in these cases the items were counted as “not mentioned”.

A large majority of the German consent documents referred to items as in section (A) such as e.g., “Research explanation and purpose” (93%) and “Biomaterial: types and quantity of specimen” (90%), to elementary conditions in (B) such as “Free and voluntary participation” (80%) or “Right to withdraw or alter consent (without disadvantage)” (97%) and to basic aspects in section (D) such as “Confidentiality of records and data/extent and limits of confidentiality” (80%) and “Privacy rights and procedures/safeguards, data processing, and identifiability of data and samples” (90%).

Information on some controversially discussed or partly not foreseeable items was given less frequently e.g., for general information (section (A)) such as “Future development and changes” (30%) or information on “Rights/Ownership of samples and data and their transfer” (57%). Also some points regarding the conditions of participation (section (B)) were considered by just half of the documents, e.g., “Decision on participation/withdrawal without affecting medical care or relationship to physician” (50%) or “Options/partial consent” (53%). Of the items regarding the consequences of participation (section (C)), the most mentioned was “Risk” (70%) and the least-mentioned “Payment/Allowance or additional costs” (33%). The mentioning of risks ranged from: no any mentioned risks at all over minimal risks for the sample/blood-taking procedure to a very detailed description of the risks using genetic data. (The latter refer to for example the knowledge as such and the related psychological burdens for the donor or family members, implication on re-identifiability of “anonymized” data, and potential implications on health insurance and employment).

Some items of presumably high importance for biobank research (in section (D) “Dealing with data and biomaterial”) were mentioned infrequently, e.g., “International Cooperation/trans-border use” (23%), “Right of access to personal data” (17%) or “Policy on use/disclosure to third parties for non-research purpose” (23%).

The number of items found also varied widely among the 30 consent documents (see **Table 2**), from a minimum of 9 (22%) to a maximum of 36 (88%). The median of items addressed across all 30 consent forms was 25.5 items (62%).

**Table 2:** Distribution of assessment items (*n* = 41, divided into five ranges) for consent content in biobank research, found in consent documents of a German biobank sample (*n* = 30)

Assessment items for consent in biobank research	Mention in German biobank consent documents	
Range	n	%
33–41 (approx. 80–100%)	3	10
25–32 (approx. 60–80%)	14	47
17–24 (approx. 40–60%)	6	20
9–16 (approx. 20–40%)	7	23
0–8 (approx. 0–20%)	0	0

## DISCUSSION AND CONCLUSION

This study illustrates that consent documents from 30 German biobanks differ widely in the range of issues that they address. Out of 41 potentially relevant issues (systematically identified in leading guidelines on consent in biomedical research) only three consent forms (10%) address more than 80% while another seven consent forms (23%) address less than 40%.

One should bear in mind that the consideration of more items does not facilitate consent or increase its validity *per se*. Nevertheless, one should have reasonable motives to minimize the extent of content and leave out relevant items.

With respect to the existing heterogeneity and different types of biobanks, it has to be considered that not all of the mentioned items must be applicable or equally relevant to each biobank. Nevertheless, core criteria for consent in biobank research and could be harmonized on an at least abstract level. Such abstract templates could then be adapted to the individual national or local context. In the German sample it is remarkable that items with a presumably high significance for biobank research in general (Cambon-Thomsen et al., 2007; Hansson, 2009; OECD, 2009; Budimir et al., 2011) were mentioned in less than 25% of all consent forms, e.g., “International Cooperation/trans-border use,” “Right of access to personal data” and “Policy on use/disclosure to third parties for non-research purpose.” The low coverage of such issues in consent forms may be partly because new developments in data protection law and research agendas are not foreseeable in detail. But it may be also because of a still limited awareness of legal, ethical, and practical challenges in biobank research and the need for their transparent communication to potential participants (Gottweis and Kaye, 2012).

Concerning the complexity of information, empirical studies need to assess which potentially relevant content issues for consent forms in biobank research can be condensed or simplified without minimizing the validity of the consent and the tissue donors’ understanding of biobank research. For a first step in this direction see Beskow (Beskow et al., 2010).

Our study has the following limitations: as a demand of further research it would be worthwhile to expand or modify the guidance-based assessment matrix for consent in biobank research in the following way: (1) to define a set of core criteria or balance the assessment items regarding their importance or relevance, (2) to outline explaining dimensions for these items, and (3) to further analyze which aspects are not covered sufficiently by the matrix respectively by the included guidances (e.g., diagnostic/therapeutic misconception). Such conceptual and empirical research would provide the basis for a deeper evaluation of existing consent documents and for the development of new consent documents. Within our explorative study of German biobank consent documents we only considered whether a potentially relevant content issue was mentioned in a consent form at all. We evaluated neither the accuracy of the explanation of this issue nor its quality, comprehensibility, or readability. Furthermore, we did not assess criteria for supportive decision-making (e.g., IPDAS-criteria IPDAS, 2005; Elwyn et al., 2006). These assessment criteria are certainly important for the development and evaluation of valid consent procedures as shown e.g., by (Beskow et al., 2010; Brehaut et al., 2012). Another limitation arises from the fact that we could only include 32% of consent forms from the full sample of 102 biobanks registered in the German Biobank Registry. However, our sample of 30 consent forms included all leading German biobanks with relatively high quality standards (including all six biobanks that are part of the National Biobank Initiative funded by the German Federal Ministry of Education and Research, BMBF). We suppose that the inclusion of further consent forms would not have changed the overall picture of the strong heterogeneity of consent forms for biobank research. Moreover, we would even expect a wider variation in content issues. Other investigations have reached similar results and support the finding that consent material in general, or in biomedical and biobank research in particular, needs improvement (Padhy et al., 2011; Pawlikowski et al., 2011; Brehaut et al., 2012; Mandava et al., 2012).

Taking into account the widely shared vision of national and international networking of biobanks and increased activities in data and sample sharing, our findings demonstrate the need of improvement and harmonization of consent procedures in the field of biobank research. Furthermore, such improvements are important to fulfil the demand of responsible biobank governance and to maintain public trust in biobank research (Gottweis and Kaye, 2012). This does also include transparent communication on consent procedures. As described in the methods section only nine consent templates (8.8%) of the 102 initially identified biobanks were available publically on the biobanks’ web sites. Furthermore, even after repeated contact and the guarantee to anonymize the results for our assessment of consent forms only 32% of registered German biobanks were willing to provide their present consent form. Beside the need to improve and harmonize consent procedures, increased transparency of such basic information should become a major aim of German biobanks, to demonstrate appropriate standards of governance.

When it comes to future improvement and harmonization of consent forms, public attitudes and expert opinion in relevant fields such as law, ethics, medicine, natural science etc. should be sought to define core criteria for consent. It may also help to overcome the lack of awareness of potentially relevant consent items and to address them in an appropriate way.

## CONCLUSION

Our findings serve as a starting point to reflect upon the spectrum of relevant consent issues in biobank research. The present study supports the systematic and transparent development of a “best practice” model of consent for biobank research. The findings show that at least the majority of consent documents in German biobanks should be improved and harmonized to better support an informed and balanced choice by potential donors and to facilitate research cooperation and networking. Further steps of such a best practice model would include the development of a best practice consent template (considering content, adherence to quality and language criteria) followed by a discussion and review of the template with other stakeholders (e.g., researchers, research ethics committees, potential biobank participants, patients’ representatives, and ethicists). Especially for a best practice model for consent forms, the understanding and validity of the text should be tested empirically (Sugarman et al., 2005; Flory et al., 2008). Finally, its use should be accompanied by continuous evaluation.

## Conflict of Interest Statement

The authors declare that the research was conducted in the absence of any commercial or financial relationships that could be construed as a potential conflict of interest.
